# Carbon emission governance and public health efficiency in transitional China: Regional differences and spatial aggregation

**DOI:** 10.1371/journal.pone.0350658

**Published:** 2026-06-02

**Authors:** Yiwen Wei, Lingxiao Guo, Qunshan Tao, Hua Wei

**Affiliations:** 1 School of Pharmaceutical Economics and Management, Anhui University of Chinese Medicine, Hefei, Anhui, China; 2 Anhui Provincial Key Laboratory of Philosophy and Social Sciences for Data Science and Innovative Development of Chinese Medicine, Hefei, Anhui, China; National Autonomous University of Mexico Institute of Geophysics: Universidad Nacional Autonoma de Mexico Instituto de Geofisica, MEXICO

## Abstract

**Background:**

With the coordinated advancement of China’s “dual carbon” goals and the Healthy China strategy, the harmonious development of carbon emission governance and public health service efficiency has emerged as a central issue in China’s sustainable transition.

**Methods:**

This study takes 31 provincial-level administrative units in China over the period 2012–2023 as the unit of analysis, and constructs a systematic analytical framework encompassing “measurement–coupling–inequality decomposition–determinant identification.” Specifically, the entropy weight method is employed to assess the composite level of carbon emission governance; the DEA-SBM model is applied to evaluate public health service efficiency; the coupling coordination degree model is used to quantify the coordinated development level of the two systems; the Dagum Gini coefficient decomposition is then utilized to identify the sources of regional disparities; the Global Moran’s Index is adopted to track the evolution of spatial agglomeration; and a two-way fixed effects Tobit model is constructed to identify associated factors.

**Results:**

The national coupling coordination degree (CCD) exhibited an overall trajectory of initial fluctuation followed by a sustained upward trend. During 2012–2017, the CCD of most provinces remained within the range of 0.44–0.63, rising broadly to the 0.54–0.72 interval after 2018. Inter-regional inequality consistently constituted the dominant source of total disparity throughout the study period. After 2018, the coordination gap between eastern and western regions continued to widen, while intra-regional disparities among western provinces simultaneously intensified, with both trends jointly driving an increasingly pronounced pattern of regional divergence. The Global Moran’s Index rose from 0.227 to 0.497, indicating a continuous strengthening of spatial agglomeration. The associated factors exhibited significant regional heterogeneity: R&D investment intensity demonstrated a significant positive association with CCD in central and western regions; urban population density exerted a significant inhibitory effect on coordination degree in western provinces; and the direction of the association between economic development level and CCD was opposite in eastern and central regions.

**Conclusions:**

Institutional time lags, factor endowment disparities, and spatial polarization mechanisms constitute the core structural barriers constraining the coordinated development of the two systems. There is an urgent need to establish a differentiated, region-specific, and phase-based policy intervention framework to promote balanced and coordinated regional development.

## 1. Introduction

Against the historical backdrop of the deep integration of ecological civilization construction and the “Healthy China” strategy, how to scientifically, efficiently, and coordinately advance the integrated development of carbon emission governance and public health service efficiency has emerged as a key issue in building a modernized environmental governance system and enhancing the quality of public services [1]. At the macro level, China, as the world’s largest carbon-emitting economy, has faced fundamental challenges to its existing economic structure and resource allocation patterns in the course of pursuing its “dual carbon” targets, as high-intensity and wide-ranging emission reduction actions have imposed profound structural pressures on established development paradigms [2]. Concurrently, environmental pollution — in particular, the environmental health risks associated with air pollution generated by high-carbon emission activities — has posed a long-term and severe threat to population health levels and the carrying capacity of the healthcare system [3]. According to data from the Ministry of Ecology and Environment, despite certain improvements in air quality, regionally concentrated and compound pollution problems remain pronounced, requiring the public health system not only to respond effectively to conventional disease burdens but also to absorb the additional health burden attributable to environmental factors [4–7]. At the policy level, the state has designated carbon emission reduction as a binding constraint target while continuously deepening healthcare system reform with the aim of improving the efficiency of medical resource allocation. The coordinated implementation of the Outline of the Healthy China 2030 Plan and the Opinions on Deeply Fighting the Pollution Prevention and Control Battle has explicitly established a policy orientation whereby environmental governance and health performance improvement must advance in tandem. Under these circumstances, how to overcome the potential contradictions between the domains of environmental governance and public health — in terms of resource investment, technological application, and policy objectives — and to achieve the coordinated development of the two systems, has become a core scientific question that warrants urgent and in-depth investigation.

Existing research has accumulated important foundations across four dimensions: carbon emission governance measurement, public health efficiency evaluation, environment-health linkages, and coupling coordination analysis.

In terms of comprehensive evaluation of carbon emission governance, scholars have progressively shifted from single emission indicators toward multidimensional composite measurements of governance performance. By integrating dimensions including emission reduction intensity, energy structure, and carbon sink capacity using the entropy weight method, existing research has systematically revealed the temporal differentiation characteristics of carbon governance levels across Chinese provinces [8]. Further research has found that eastern provinces have continuously widened the governance performance gap with western regions by virtue of their first-mover advantage in industrial green transformation [9–11]. These studies collectively indicate that regional differentiation in carbon emission governance levels has significant structural roots, and that the selection of different indicator dimensions directly influences the assessment of inter-provincial disparity patterns.

With respect to public health service efficiency measurement, the SBM model has been employed to identify dual efficiency losses in western regions characterized by the simultaneous presence of input redundancy and output shortfall [12]. Building on this, subsequent research incorporated undesirable outputs to reveal the suppression mechanism through which healthcare resource misallocation constrains overall health system efficiency [13]. Existing studies have broadly confirmed the significant regional imbalance in public health efficiency in China; however, the systemic causes underlying these efficiency disparities remain insufficiently explored, particularly with regard to the structural influence of external systems — such as carbon emission governance — which has yet to be adequately examined.

Regarding the association between environmental governance and health outcomes, a number of scholars have estimated the negative impact of air pollution on life expectancy at birth through quasi-natural experimental designs [14–18], empirically verifying from the health outcome perspective the substantive influence of environmental quality on population health. Building on these findings, subsequent research has further verified the transmission pathway through which environmental regulation indirectly improves public health efficiency by reducing pollution emissions, providing preliminary mechanism-level evidence for understanding the association between carbon emission governance and health system efficiency [19,20]. Nevertheless, the aforementioned studies predominantly focus on unidirectional influences in a single direction, and have not yet incorporated both systems into a unified evaluative framework for coordinated development.

In the domain of coupling coordination analysis, the coupling coordination degree model has been applied to the collaborative analysis of green development and healthcare systems [21–24], while other work has constructed a coordinated development index for environmental and health systems based on panel data [25,26]. With respect to spatial effects and determinant factors, significant spatial agglomeration characteristics have been identified in the efficiency of healthcare resource allocation in China [27–29], and R&D investment has been confirmed to exert a significant positive effect on the coordination degree of environment-health systems [30,31].

In summary, existing research remains insufficient in the systematic integration of carbon emission governance and public health efficiency, as well as in the in-depth analysis of regional coordination mechanisms — in particular, there is a notable absence of systematic empirical investigation into the spatial differentiation patterns of coordinated development between the two systems and the regional heterogeneity of their underlying associational logic. Against this backdrop, as the “dual carbon” goals and the Healthy China strategy are advanced in tandem, the potential tensions between the two systems in terms of resource allocation, policy objectives, and institutional design have become increasingly pronounced, underscoring the urgent need to clarify the regional differentiation patterns of their coordinated development and to identify their core associated factors. Through the construction of a systematic analytical framework encompassing “measurement–coupling–inequality decomposition–determinant identification,” this study identifies a dual polarization pattern in the coordinated development of carbon emission governance and public health efficiency across Chinese provinces — characterized by the continuous widening of the coordination gap between eastern and western regions and the simultaneous intensification of intra-regional disparities within western provinces after 2018 — within which inter-regional inequality consistently dominates total imbalance, thereby advancing beyond the generalized treatment of regional disparity sources in existing literature. Concurrently, through spatial agglomeration analysis and regionally heterogeneous association analysis, the study reveals the deep-seated structural contradiction whereby a uniform national policy framework cannot effectively respond to the distinct structural constraints of different regions. As the world’s largest carbon-emitting economy, China’s experience of regional polarization patterns and institutional time lag effects in the coordinated pursuit of carbon neutrality goals and public health efficiency improvement offers transferable empirical evidence and policy insights for other developing countries and emerging economies undergoing green transitions, facilitating their understanding of the inherent tensions in the coordinated development of environmental governance and health systems.

## 2. Coupling coordination mechanism between carbon emission governance and public health service efficiency

**Fig 1:**
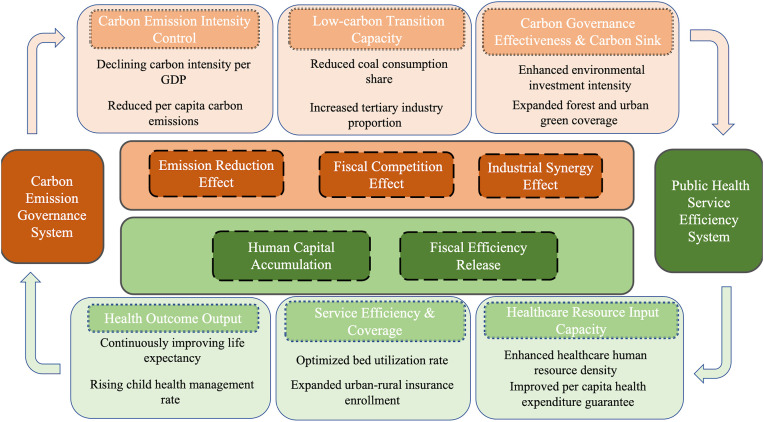
Coupling coordination theoretical model.

Building on the review of existing literature, this study further constructs a bidirectional theoretical transmission mechanism between carbon emission governance and public health service efficiency, with the aim of clarifying the intrinsic logic of the co-evolutionary dynamics of the two systems and providing a theoretical foundation for the subsequent empirical analysis (Fig 1).

### 2.1. Multidimensional transmission mechanisms through which carbon emission governance acts upon public health service efficiency

#### 2.1.1. Compression of total pollutant emissions driving sustained improvements in residents’ health outcomes.

The sustained decline in carbon emission intensity — measured by carbon emissions per unit of GDP and per capita carbon emissions — directly compresses the total volume of atmospheric pollutant emissions resulting from fossil fuel combustion. The reduction in the share of coal consumption within the energy structure further decreases the concentration of harmful substances such as PM2.5 and SO₂, thereby lowering the morbidity and mortality rates of pollution-related diseases, including respiratory diseases and cardiovascular and cerebrovascular diseases. Within the public health efficiency indicator framework, life expectancy at birth and the health management rate for children under seven years of age serve as core output indicators reflecting residents’ health outcomes. A reduction in the disease burden attributable to pollution will directly improve performance on these output indicators, thereby driving efficiency gains under conditions of unchanged input levels. Accordingly, improvements in emission intensity on the carbon governance side may indirectly act upon the output efficiency of the public health system through the structural optimization of health demand.

#### 2.1.2. Rigid emission reduction constraints inducing competitive fiscal allocation between environmental expenditure and health investment.

The environmental pollution control investment intensity within the composite carbon emission governance indicator system reflects the magnitude of local government financial investment in pollution control. Under conditions of finite local fiscal capacity, the expansion of environmental expenditure driven by binding emission reduction targets will generate a competitive allocation relationship with per capita healthcare expenditure. For central and western provinces characterized by relatively low fiscal self-sufficiency, the crowding-out effect of environmental expenditure will directly compress the fiscal space available for public health service investment, thereby constraining improvements in output indicators such as bed occupancy rate and medical insurance enrollment rate. The existence of this pathway implies that the relationship between carbon governance and public health efficiency is not purely positive; the resource competition mechanism may produce stage-specific negative effects under particular fiscal conditions. This constitutes one of the core reasons why this study adopts the coupling coordination degree rather than simple correlation indicators to evaluate the relationship between the two systems.

#### 2.1.3. Low-carbon industrial transition synergistically expanding the supply foundation and efficiency space of public health services.

The share of tertiary industry value-added in the low-carbon transition capacity indicators measures the degree to which regional industrial structure is upgrading toward a service-oriented direction. A service-sector-dominated industrial structure simultaneously reduces carbon emission intensity per unit of GDP and, by expanding the scale of modern service industries such as healthcare and health management, objectively broadens the supply foundation of public health services. This contributes to enhancing the resource density of input indicators such as the number of primary healthcare institutions per 10,000 population and promotes improvements in health service efficiency through market competition mechanisms. Concurrently, improvements in clean production levels — as represented by the increase in the comprehensive utilization rate of general industrial solid waste — also indirectly optimize the input-output structure of the public health system by reducing the “passive consumption” of medical resources attributable to industrial pollution.

### 2.2. Reverse reinforcement mechanisms through which public health service efficiency acts upon carbon emission governance capacity

#### 2.2.1. Improvements in residents’ health levels consolidating the human capital foundation for green and low-carbon transition.

Residents’ health levels — as represented by output indicators such as life expectancy at birth and child health management rates — constitute an important dimension of regional human capital quality. Efficient public health services provide sustained and stable human capital support for low-carbon industrial transition and green technology research and development by extending healthy working years and reducing productivity losses attributable to illness. Within the carbon governance indicator system, R&D-driven green innovation capacity and the clean structural transformation of industry are both highly dependent on the continuous supply of healthy labor. Accordingly, improvements in public health efficiency create conditions for the deepening of carbon emission governance from the human capital supply side.

#### 2.2.2. Optimization of health fiscal efficiency releasing resource space to support carbon emission governance investment capacity.

Improvements in public health service efficiency imply the achievement of higher levels of health output under existing input scales, thereby reducing medical resource waste and fiscal overexpenditure attributable to inefficiency. The increase in urban and rural residents’ medical insurance enrollment rates contributes to reducing the risk of poverty due to illness among residents, alleviating the fiscal pressure on government-funded medical assistance programs, and objectively creating additional fiscal space for local governments to direct toward environmental pollution control investment. This pathway is of particular importance for central and western provinces characterized by pronounced fiscal constraints, and represents a key mechanism for understanding how improvements in the coordination level of the two systems are mutually constitutive preconditions.

### 2.3. Bidirectional interaction mechanisms and coupling coordination logic between carbon emission governance and public health service efficiency

The interaction between carbon emission governance and public health service efficiency is not unidirectional or linear, but rather exhibits the characteristics of a complex system in which multiple pathways coexist, directional influences are mutually intertwined, and interaction intensity varies according to regional conditions. From the perspective of forward pathways, the reduction of emission pressure on the carbon governance side may enhance health system output efficiency by improving residents’ health outcomes, and the service-oriented upgrading of industrial structure may generate synergies with the expansion of public health services. However, the expansion of environmental fiscal expenditure driven by rigid emission reduction constraints may crowd out health investment under conditions of limited fiscal capacity, producing inhibitory effects that run counter to the logic of synergy. From the perspective of reverse pathways, improvements in public health efficiency feed back into carbon governance capacity through human capital accumulation and the release of fiscal efficiency gains; however, this transmission is equally subject to the constraints of regional development stages and institutional environments, and cannot be effectively activated in all provinces. From the perspective of long-term sustainability, the deepening of carbon emission governance depends on the continuous supply of healthy labor and broad social endorsement of green transition; conversely, if environmental governance is persistently compromised, the accumulation of environmental health risks will impose an increasing disease burden on the health system, generating efficiency losses of greater magnitude. This dual attribute — characterized by the simultaneous presence of synergistic potential and competitive tension — determines that the relationship between the two systems is highly context-dependent, contingent on regional factor endowments, fiscal structures, and levels of policy coordination, and cannot be adequately characterized by simple notions of positive correlation or zero-sum trade-off. In light of this, the present study adopts the coupling coordination degree as the core measurement instrument, simultaneously capturing the intensity of inter-system interaction and the overall development level of both systems, thereby transforming the complex bidirectional relationship described above into a quantifiable and comparable evaluative dimension of coordinated development.

## 3. Methodology and indicator selection

### 3.1. Evaluation and coupling

#### 3.1.1. Entropy weight method.

As the first step of the analytical framework, this study requires the transformation of multidimensional carbon emission governance behaviors into a composite quantitative index that can be systematically compared with public health service efficiency. The comprehensive level of carbon emission governance encompasses eight indicators across four dimensions — emission intensity, low-carbon transition capacity, governance effectiveness, and carbon sink capacity. Given that these indicators differ substantially in units of measurement and their relative importance cannot be reliably determined through prior knowledge, the entropy weight method assigns objective weights based on the actual degree of variation in the indicator data, thereby effectively avoiding the bias inherent in subjective weighting approaches. This method is well-suited for the composite integration of multidimensional governance performance, and is therefore employed to calculate the comprehensive carbon emission governance level index U for each province, providing one of the core input variables for the subsequent construction of the coupling coordination degree model [32]. In this study, the entropy weight method is applied to measure the comprehensive level of carbon emission governance across Chinese provinces, where the composite index is constituted by indicators across multiple dimensions, including emission reduction intensity, technological investment, and policy effectiveness. The degree of dispersion among indicators is reflected by computing the information entropy of each indicator: the greater the degree of variation in an indicator — that is, the smaller its information entropy — the greater the amount of information it carries, and accordingly, the higher the weight assigned to it. The specific calculation procedure is as follows:

(1) Data Standardization: Given the differences in units of measurement and ranges of variation across indicators, the raw data must first be standardized to eliminate the influence of dimensional discrepancies. For positive and negative indicators, the following formulas are applied respectively. Let $x_{ij}$ denote the raw value of province *i* on indicator *j*; the standardized value $y_{ij}$ is computed as:


$$\begin{array}{c} \begin{array}{c} \text{Positive indicator}:y_{ij}=\frac{x_{ij}-\text{min}(x_{j})}{\text{max}(x_{j})-\text{min}(x_{j})} \end{array} \end{array}$$ (1)

$$\begin{array}{c} \begin{array}{c} \text{Negative indicator}:y_{ij}=\frac{\text{max}(x_{j})-x_{ij}}{\text{max}(x_{j})-\text{min}(x_{j})}\; \end{array} \end{array}$$ (2)

where $\text{max}(x_{j})$ and $\text{min}(x_{j})$ denote the maximum and minimum values of indicator *j* across all provinces, respectively. To avoid undefined logarithmic operations, a translation adjustment is applied to the standardized values: $Y_{ij}=y_{ij}+\in$, where ∈ is a sufficiently small positive constant.

(2) Calculation of Indicator Proportions. The proportion $P_{ij}$ of province *i* under indicator *j* is computed as:


$$\begin{array}{c} \begin{array}{c} P_{ij}=\frac{Y_{ij}}{\sum_{i=\text{1}}^{n}Y_{ij}} \end{array} \end{array}$$ (3)

(3) Calculation of Information Entropy. The information entropy $e_{j}$ of indicator *j* is calculated as:


$$\begin{array}{c} \begin{array}{c} e_{j}=-\frac{\text{1}}{\text{ln}(n)}\sum_{i=\text{1}}^{n}P_{ij}\;\text{ln}\;(P_{ij})\; \end{array} \end{array}$$ (4)

where $\;\text{0}\leq e_{j}\leq \text{1}$.

(4) Calculation of Indicator Weights. The objective weight $W_{j}$ of indicator *j* is derived from its information entropy as:


$$\begin{array}{c} \begin{array}{c} W_{j}=\frac{\text{1}-e_{j}}{\sum_{j=\text{1}}^{m}(\text{1}-e_{j})}\; \end{array} \end{array}$$ (5)

where *m* denotes the total number of indicators, satisfying $\sum_{j=\text{1}}^{m}W_{j}=\text{1}$.

#### 3.1.2. DEA-SBM model.

Building upon the measurement of the comprehensive level of carbon emission governance, the second step of the analytical framework requires an independent assessment of public health service efficiency in order to obtain the other core input variable required for the coupling coordination degree model. The public health service system is characterized by a complex structure involving multiple inputs and multiple outputs, and it is difficult to obtain unified market price information for the monetary conversion of input and output factors. By directly incorporating input redundancy and output shortfall into the objective function, the SBM model is capable of more accurately reflecting the sources of efficiency loss within the public health service system [33], thereby providing a reliable efficiency measurement foundation for evaluating the coordinated development of carbon emission governance and public health efficiency.


$$\begin{array}{c} \begin{array}{c} \text{min}\rho =\frac{\text{1}-\frac{\text{1}}{m}\sum_{i=\text{1}}^{m}\frac{\overset{-}{\underset{i}{s}}}{x_{ik}}}{\text{1}+\frac{\text{1}}{q}\sum_{r=\text{1}}^{q}\frac{\overset{+}{\underset{r}{s}}}{y_{rk}}} \end{array} \end{array}$$ (6)

where ${\text{x}}_{\text{ik}}$ and ${\text{y}}_{\text{rk}}$ represent the i-th input and r-th output of the K-th decision-making unit (DMU), respectively; $\overset{-}{\underset{\text{i}}{\text{s}}}$ and $\overset{+}{\underset{\text{r}}{\text{s}}}$ denote input redundancy and output shortfall, respectively; and m and q represent the numbers of input and output indicators, respectively. When ρ = 1, the DMU lies on the production frontier and is DEA-efficient; when ρ < 1, efficiency loss is present. Based on the operational characteristics of the public health service system, an evaluation framework comprising 5 input indicators and 4 output indicators is constructed. The SBM model possesses desirable properties of units invariance and monotonicity, enabling effective efficiency evaluation of indicators with heterogeneous units of measurement, making it well-suited for the measurement requirements of this study.

#### 3.1.3. Coupling coordination degree model.

Having obtained the comprehensive level of carbon emission governance and public health service efficiency independently, the analytical framework proceeds to its third step: integrating the two separate measures into a composite evaluation index that reflects the synergistic state of the two systems. Examining the absolute levels of either system in isolation is insufficient to determine whether the two systems are in a state of benign coordination. To address this, the present study introduces the coupling coordination degree model, which simultaneously captures the intensity of interaction between the two systems (coupling degree C) and the overall development level of both systems (composite evaluation index T), thereby transforming the complex bidirectional relationship into an objectively quantifiable and comparable dimension for evaluating coordinated development. This provides a unified analytical object for the subsequent regional disparity decomposition and spatial analysis [34].

(1) Coupling Degree

The coupling degree C reflects the intensity of interaction between the two systems, and is calculated using the following formula:


$$\begin{array}{c} \begin{array}{c} C=\sqrt{\frac{U\cdot E}{(U+E{)}^{\text{2}}}} \end{array} \end{array}$$ (7)

where U denotes the composite carbon emission governance level index derived from the entropy weight method, and E denotes the public health service efficiency value obtained from the DEA-SBM model; both are standardized to the interval [0, 1]. The value of C ranges from 0 to 1; a higher C value indicates stronger interaction between the two systems and closer systemic association.

(1) Comprehensive Evaluation Index. The coupling degree C alone is insufficient to distinguish whether the two systems are in a state of virtuous mutual promotion or low-level equilibrium. A comprehensive evaluation index T is therefore introduced to reflect the overall development level of the two systems:


$$\begin{array}{c} \begin{array}{c} T=\alpha S+\beta E \end{array} \end{array}$$ (8)

where α and β represent the weight coefficients assigned to the composite carbon emission governance level and public health service efficiency, respectively. Given that this study regards both environmental governance and public health as equally important objectives for sustainable development, the weights are set as α = β = 0.5.

(2) Coupling Coordination Degree. Building upon the coupling degree C and the comprehensive evaluation index T, the coupling coordination degree D is constructed as:


$$\begin{array}{c} \begin{array}{c} D=\sqrt{C\times T} \end{array} \end{array}$$ (9)

The value of D ranges from 0 to 1; a higher D value indicates a higher level of synergistic development between the two systems and a greater tendency toward a virtuous coordination state. Following the classification scheme established in the existing literature [35], D values are divided into 10 grades (Table 1) to facilitate intuitive assessment of the coordinated development stage of each province during the study period.

**Table 1:** Classification criteria for the coupling coordination degree between carbon emission governance and public health efficiency in China.

Coupling Coordination Degree (D)	Interval Type	Coupling and Coordination Level
[0.00–0.10)	Disordered	Extreme disorder
[0.10–0.20)		Severe disorder
[0.20–0.30)		Moderate disorder
[0.30–0.40)		Mild disorder
[0.40–0.50)	Running-in	Near disorder
[0.50–0.60)		Barely coordinated
[0.60–0.70)	Synergistic	Primary coordination
[0.70–0.80)		Intermediate coordination
[0.80–0.90)		Good coordination
[0.90–1.00]		High-quality coordination

### 3.2. Regional evolution and spatiotemporal analysis

#### 3.2.1. Dagum Gini coefficient.

Taking the coupling coordination degree as the analytical object, the fourth step of the framework shifts toward a refined identification of the structural sources of regional disparities. The overall Gini coefficient can only measure the degree of aggregate inequality, and is incapable of addressing the policy-critical question of whether disparities primarily originate from differences between regions or from within regions [36]. By introducing the concept of hypervariable density, the Dagum Gini coefficient decomposes overall disparity into three sources — intra-regional difference, inter-regional net difference, and hypervariable density — thereby effectively resolving the problem of sub-sample cross-overlap. This approach enables a structural tracking of the sources of spatial inequality in the coupling coordination degree, and provides empirical evidence for the precise design of differentiated regional policies [37].

The Dagum Gini coefficient is defined as:


$$\begin{array}{c} \begin{array}{c} G=\frac{\sum_{j=\text{1}}^{k}\sum_{h=\text{1}}^{k}\sum_{i=\text{1}}^{n_{j}}\sum_{r=\text{1}}^{n_{h}}\left|D_{ji}-D_{hr}\right|}{\text{2}n^{\text{2}}\bar{D}} \end{array} \end{array}$$ (10)

where *G* denotes the overall national Dagum Gini coefficient; *k* represents the number of regional divisions (i.e., eastern, central, and western regions, k = 3); *n* is the total number of provinces; $n_{j}$ and $n_{\text{h}}$ are the number of provinces in the *j*-th and h-th regions, respectively; $D_{ji}$ and $D_{\text{h}r}$ represent the coupling coordination degree of the *i*-th province in region *j* and the *r*-th province in region *h*, respectively; and $\bar{D}$ denotes the national mean coupling coordination degree. According to Dagum’s theoretical framework, the overall Gini coefficient *G* is decomposed into three components:


$$\begin{array}{c} \begin{array}{c} G=G_{w}+G_{nb}+G_{t} \end{array} \end{array}$$ (11)

$$\begin{array}{c} \begin{array}{c} G_{w}=\sum_{j=\text{1}}^{k}G_{jj}P_{j}\rho_{j} \end{array} \end{array}$$ (12)

$$\begin{array}{c} \begin{array}{c} G_{nb}=\sum_{j=\text{1}}^{k}\sum_{h=\text{1},h=\;\;/\;j}^{k}G_{jh}\left(P_{j}\rho_{h}+P_{h}\rho_{j}\right) \end{array} \end{array}$$ (13)

where $G_{w}$ denotes the contribution of intra-regional differences (Intra-regional Difference Contribution), representing the weighted average of disparities among provinces within each region; $G_{nb}$ represents the contribution of inter-regional net differences (Inter-regional Net Difference Contribution), reflecting the disparities attributable to differences in mean coupling coordination degrees across regions; and $G_{t}$ denotes the contribution of hypervariable density (Hypervariable Density Contribution), capturing the cross-regional overlap in coupling coordination degrees—that is, the disparities arising from cross-regional interaction effects.

#### 3.2.2. Moran’s index.

The decomposition of the sources of regional disparities reveals the static distributional pattern of the coupling coordination degree. The fifth step of the framework further inquires whether there exists systematic spatial dependence in the level of coordinated development among adjacent provinces. To address this question, the present study employs the Global Moran’s Index to examine the spatial autocorrelation characteristics of the coupling coordination degree, dynamically tracking the evolutionary trends of spatial agglomeration patterns throughout the study period, and assessing whether provinces with high values and those with low values each exhibit tendencies toward geographic clustering [38,39]. This provides a basis for understanding the spatial reinforcement mechanism underlying regional differentiation, and lays the foundation for interpreting spatial spillover effects in the subsequent analysis of influencing factors.

The Global Moran’s Index is calculated as:


$$\begin{array}{c} \begin{array}{c} I=\frac{n\sum_{i=\text{1}}^{n}\sum_{j=\text{1}}^{n}w_{ij}(x_{i}-\bar{x})(x_{j}-\bar{x})}{\sum_{i=\text{1}}^{n}\sum_{j=\text{1}}^{n}w_{ij}\sum_{i=\text{1}}^{n}(x_{i}-\bar{x}{)}^{\text{2}}} \end{array} \end{array}$$ (14)

where *I* is the Global Moran’s Index; *n* is the total number of spatial units (30 provinces in this study); $x_{i}$ and $x_{j}$ represent the coupling coordination degree values of provinces *i* and j, respectively; $\bar{x}$ is the mean coupling coordination degree across all provinces; and $w_{ij}$ is the spatial weight between provinces *i* and j in the spatial weight matrix. This study adopts theGeographic Contiguity Matrix, whereby $w_{ij}$ = 1 if province *i* and province j are geographically adjacent, and $w_{ij}$ = 0 otherwise.

The Global Moran’s Index ranges from −1–1. When I > 0, the coupling coordination degree exhibits positive spatial autocorrelation, indicating that high-value regions tend to be adjacent to other high-value regions and low-value regions cluster together, reflecting spatial agglomeration. When I < 0, negative spatial autocorrelation is present, whereby high-value and low-value regions alternate in spatial distribution. When I approaches 0, the coupling coordination degree follows a random spatial distribution with no significant spatial association. By computing the Global Moran’s Index for each year from 2012 to 2023, the evolutionary trajectory of the spatial agglomeration pattern of the coupling coordination degree can be dynamically tracked.

### 3.3. Two-way fixed effects tobit regression

Upon completing the measurement of the coupling coordination degree, the decomposition of regional disparities, and the identification of spatial agglomeration patterns, the final step of the analytical framework turns to the exploration of causal mechanisms: what factors drive the level and variation of the coupling coordination degree across provinces? As the dependent variable, the coupling coordination degree exhibits a two-sided censored characteristic (0 ≤ CCD ≤ 1), and this statistical property necessitates the preferential adoption of a Tobit framework capable of handling censored data. Standard spatial econometric models — such as the Spatial Durbin Model and the Spatial Error Model — assume by default that the dependent variable is continuous and unbounded; forcibly incorporating a spatial weight matrix while disregarding the censored nature of the dependent variable would likewise produce biased estimates, representing a fundamental methodological incompatibility [40–42]. Furthermore, spatial Tobit models capable of simultaneously handling two-sided censoring and two-way fixed effects remain methodologically immature in the existing literature, and their implementation via available software is subject to considerable limitations. Based on the foregoing considerations, this study adopts the two-way fixed effects Tobit model as the benchmark framework for identifying influencing factors. The model specification is as follows:


$$\begin{array}{c} \begin{array}{c} CC\overset{*}{\underset{it}{D}}=\alpha +\beta X_{it}+\gamma_{i}+\delta_{t}+\varepsilon_{it} \end{array} \end{array}$$ (15)

$$\begin{array}{c} CCD_{it}=\left\{ \begin{array}{c} @l\text{0},\;if\;CC\overset{*}{\underset{it}{D}}\leq \text{0}\\ CC\overset{*}{\underset{it}{D}},\;if\;\text{0}<CC\overset{*}{\underset{it}{D}}<\text{1}\\ \text{1},\;if\;CC\overset{*}{\underset{it}{D}}\geq \text{1} \end{array}\right.\; \end{array}$$

where $CC\overset{*}{\underset{it}{D}}$ is the latent variable representing the potential coupling coordination level between carbon emission governance and public health efficiency in province *i* in year *t*; $CCD_{it}$ is the observed value; $X_{it}$ is a vector of explanatory variables including economic developmen*t* level, industrial structure, technological innovation, environmental regulation, fiscal expenditure, and population density; $\gamma_{i}$ denotes individual fixed effects, controlling for time-invariant heterogeneity across provinces; $\delta_{t}$ represents time fixed effects, controlling for common time trends affecting all provinces; and $\epsilon_{it}$ is the stochastic error term assumed to follow a normal distribution, $\epsilon_{it}\sim N(\text{0},\sigma^{\text{2}})$. Model parameters are estimated using the maximum likelihood estimation (MLE) method. Regarding variable processing, given the large cross-provincial variation in per capita GDP and the proportion of elderly population, both variables are standardized using Z-score normalization to eliminate dimensional interference with coefficient estimation. Urban population density, which exhibits a highly right-skewed distribution across provinces, is log-transformed prior to inclusion in the regression to mitigate the influence of extreme values. Urbanization rate and R&D expenditure intensity, already expressed as ratios, are incorporated directly into the model.

### 3.4. Indicator selection

#### 3.4.1. Carbon emission governance indicators.

Drawing on a review of existing literature on frameworks for measuring the level of carbon emission governance, this study constructs a comprehensive indicator system for carbon emission governance from four dimensions: carbon emission intensity, low-carbon transition capacity, carbon governance effectiveness, and carbon sink capacity (see Table 2). On the emission side, carbon emissions per unit of GDP and per capita carbon emissions are selected as proxy indicators for carbon emission intensity; the former reflects the carbon efficiency of economic activities, while the latter captures the emission pressure from a demographic perspective, together characterizing the dual economic-demographic intensity of regional carbon emissions [43]. On the transition side, the share of coal consumption is used to measure the degree of clean energy structure transformation, and the share of value added by the tertiary industry is employed to represent the level of service-oriented upgrading of the industrial structure, both of which align with China’s low-carbon development strategy centered on the dual axes of energy structure adjustment and industrial transformation [44]. On the governance side, the intensity of investment in environmental pollution control and the comprehensive utilization rate of general industrial solid waste are introduced; the former measures the extent of financial commitment by local governments to pollution control, while the latter reflects the degree of cleaner production in industrial processes, and together they characterize the implementation effectiveness of carbon governance policies [45]. On the carbon sink side, forest coverage rate and the green coverage rate of built-up areas are incorporated to assess a region’s carbon absorption and sequestration capacity from the dual perspectives of natural carbon sinks and urban green spaces [46]. The indicator system systematically and objectively reflects the comprehensive level of each province across the full chain of carbon emission governance, encompassing four dimensions: emission pressure, transition pathways, governance investment, and ecological compensation.

**Table 2:** Indicator system for carbon emission governance level.

Primary Indicator	Secondary Indicator	Tertiary Indicator	Unit	Direction
Carbon Emission Intensity	Economic carbon emission efficiency	Carbon emissions per unit GDP	tonnes/10,000 CNY	–
	Demographic carbon emission intensity	Per capita carbon emissions	tonnes/person	–
Low-Carbon Transition Capacity	Energy structure optimization	Share of coal consumption	%	–
	Industrial structure upgrading	Share of tertiary industry value-added	%	+
Carbon Governance Effectiveness	Pollution control investment	Environmental pollution control investment intensity	%	+
	Industrial cleaner production	omprehensive utilization rate of general industrial solid waste	%	+
Carbon Sink Capacity	Ecological carbon sink	Forest coverage rate	%	+
		Urban green coverage rate	%	+

#### 3.4.2. Public health service efficiency indicator system.

In accordance with the dual requirements of the DEA-SBM model for input and output indicators, this study constructs a public health service efficiency evaluation indicator system encompassing three categories of input dimensions — human resources, physical resources, and financial resources — and three categories of output dimensions — health outcomes, service efficiency, and service coverage (see Table 3). On the input side, the number of licensed (assistant) physicians per thousand population and the number of registered nurses per ten thousand population are used to characterize the supply density of human resources; the number of medical institution beds per ten thousand population and the number of primary healthcare institutions per ten thousand population are employed to measure the allocation level of physical resources; and per capita healthcare expenditure is incorporated to reflect the intensity of financial resource investment. The combination of these three categories of input indicators systematically covers the core elements of the supply side of public health services [47–49]. On the output side, life expectancy per capita is used to reflect the ultimate health outcome of healthcare services; bed utilization rate is employed to measure the actual conversion efficiency of physical resources; and the urban-rural residents’ basic medical insurance enrollment rate, neonatal visit rate, and health management rate for children under seven years of age are jointly used to characterize the breadth of population coverage and the degree of implementation of basic public health services. The inclusion of the latter three indicators is particularly consistent with China’s healthcare reform policy orientation of “strengthening primary-level institutions and emphasizing prevention,” and is capable of effectively capturing the efficiency improvements arising from the extension of public health services from the curative end to the preventive end [50–52]. Under the premise of balancing data availability and indicator representativeness, this input-output indicator system is able to comprehensively reflect the actual operational efficiency of provincial-level public health service systems, thereby providing a reliable efficiency measurement foundation for the subsequent coupling coordination analysis with the level of carbon emission governance.

**Table 3:** Indicator system for public health service efficiency.

Dimension		Indicator	Unit
Input Indicators	Human resource input	Licensed (assistant) physicians per thousand population	persons/1,000
		Registered nurses per ten thousand population	persons/10,000
	Physical resource input	Medical institution beds per ten thousand population	beds/10,000
		Primary healthcare institutions per ten thousand population	institutions/10,000
	Financial resource input	Per capita healthcare expenditure	CNY/person
Output Indicators	Health outcome	Per capita life expectancy	years
	Service efficiency	Bed occupancy rate	%
	Service coverage	Urban–rural resident medical insurance enrollment rate	%
		Neonatal home visit rate	%
		Health management rate for children under 7 years of age	%

#### 3.4.3. Influencing factor indicators.

This study selects five indicators from four dimensions — economic development, social structure, demographic structure, and environmental regulation — as the influencing factors of the coupling coordination degree between carbon emission governance and public health efficiency (see Table 4). Per capita GDP, as the core proxy indicator of economic development, not only constitutes the material foundation upon which local governments advance carbon emission governance and healthcare service investment, but also directly determines the fiscal carrying capacity of both systems under conditions of resource competition [53]. The urbanization rate is introduced from the perspective of social structure; the urbanization process, on the one hand, drives adjustments in energy consumption structure and the spatial agglomeration of carbon emissions, and on the other hand, reshapes the demand scale and supply mode of public health services, making it a key social transformation variable affecting the coordinated development pattern of the two systems [54]. The proportion of the population aged 65 and above reflects the degree of aging in the regional demographic structure; the deepening of population aging intensifies the carrying pressure on the public health service system while also indirectly influencing carbon emission governance levels through its effects on labor force structure and industrial energy consumption patterns. Its inclusion in the model enables the identification of the compound influence pathways through which demographic structural change affects the coupling coordination degree [55]. R&D expenditure intensity, measured by the ratio of regional research and development expenditure to GDP, captures technological innovation capacity; the research, development, and diffusion of green and low-carbon technologies constitute the endogenous driving force behind the simultaneous improvement of carbon emission reduction and healthcare service efficiency, and this indicator is capable of effectively capturing the promotional effect of technological progress on the coordinated development of the two systems [56]. Urban population density, represented by the number of urban residents per unit of administrative area, characterizes the degree of spatial agglomeration; the spatial distribution pattern of the population both directly affects the supply radius and resource utilization efficiency of public health services, and influences carbon governance performance through changes in energy consumption intensity and transportation emission levels. Its incorporation into the spatial structure dimension facilitates the identification of structural constraints on regional coordinated development from the perspective of spatial carrying capacity.

**Table 4:** Indicator system for influencing factors.

Dimension	Indicator	Symbol	Description	Unit
Economic development	Per capita GDP	GDPpc	Gross domestic product divided by the total population of the region	10,000 CNY/person
Social structure	Urbanization rate	UrbR	Percentage of urban population in the total population	%
Demographic structure	Population aged 65 and above	Pop65+	Proportion of population aged 65 and above in the total population	%
Environmental regulation	R&D expenditure intensity	R&DInt	Ratio of R&D expenditure to regional GDP	%
	Urban population density	PopDen	Urban population per unit administrative area	persons/km²

### 3.5. Data sources

The data employed in this study cover 31 provincial-level administrative units in China (excluding Hong Kong, Macao, and Taiwan) over the study period from 2012 to 2023, constituting a balanced panel dataset. The data sources for the comprehensive carbon emission governance indicator system are as follows: data on carbon emissions per unit of GDP and per capita carbon emissions are sourced from the *China Energy Statistical Yearbook* and CEADs (Carbon Emission Accounts and Datasets for China); data on the share of coal consumption are sourced from the *China Energy Statistical Yearbook;* data on the share of value added by the tertiary industry are sourced from the *China Statistical Yearbook*; data on the intensity of investment in environmental pollution control are sourced from the *China Environment Statistical Yearbook*; data on the comprehensive utilization rate of general industrial solid waste are sourced from the *China Environment Statistical Yearbook*; and data on forest coverage rate and the green coverage rate of built-up areas are sourced from the *China Forestry Statistical Yearbook* and the statistical bulletins on national economic and social development of each province. All data for the public health service efficiency indicator system are sourced from the *China Health Statistics Yearbook*. Data on per capita GDP, urbanization rate, and the proportion of the population aged 65 and above are sourced from the *China Statistical Yearbook* and the statistical yearbooks of each province; data on R&D expenditure intensity are sourced from the *China Science and Technology Statistical Yearbook*; and data on urban population density are derived from the urban population figures and administrative area measurements recorded in the *China Urban Statistical Yearbook.*

### 3.6. Ethics statement

This study is based entirely on publicly available aggregated statistical data obtained from official Chinese government yearbooks and databases. No human participants, animal subjects, or personally identifiable information were involved in this research. Therefore, ethical approval was not required.

## 4. Results

### 4.1. Comprehensive level of carbon emission governance

**Fig 2:**
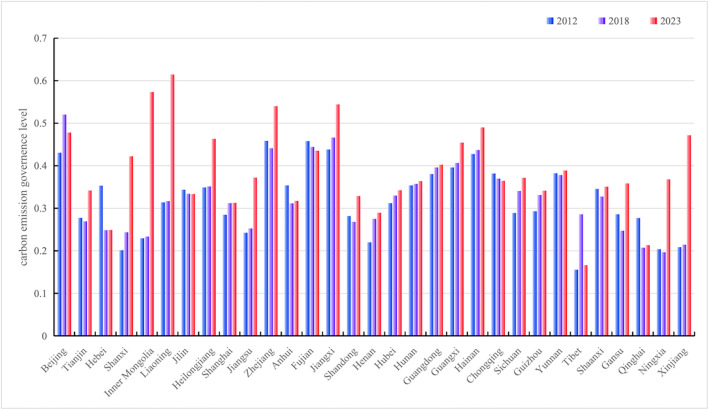
Comprehensive level of carbon emission governance in China, 2012, 2018 and 2023.

The comprehensive level of carbon emission governance in China, as measured by the entropy weight method, exhibits pronounced spatiotemporal evolutionary characteristics and regional differentiation patterns (see Fig 2). In terms of temporal evolution, the overall level of carbon emission governance nationwide demonstrated an upward trend during the period from 2012 to 2023, although the magnitude of improvement varied significantly across provinces. In 2012, the governance levels of most provinces were generally concentrated in the range of 0.2 to 0.5, with municipalities such as Beijing, Shanghai, and Tianjin at relatively higher levels (approximately 0.5), while western provinces such as Qinghai, Tibet, and Xinjiang all fell below 0.3. By 2018, regional differentiation became more pronounced, with the governance levels of eastern coastal provinces such as Zhejiang, Fujian, and Jiangsu surging to above 0.6, while the western region remained at the relatively low level of 0.2 to 0.3. The spatial pattern in 2023 indicates that high-value areas expanded further and extended toward the central region, with Fujian, Zhejiang, and Hainan reaching the highest level of above 0.6, forming a high-value agglomeration belt along the southeastern coast; central provinces such as Hunan, Hubei, and Anhui improved to the range of 0.3 to 0.4; while northwestern provinces such as Qinghai, Xinjiang, and Gansu remained at low levels of approximately 0.2. In terms of spatial distribution, a pronounced gradient pattern of “higher in the east and lower in the west, decreasing from coastal to inland areas” is evident, and the gap in governance levels among the three major eastern, central, and western regions persisted throughout the observation period with a tendency toward widening.

### 4.2. Spatial evolution pattern of public health service efficiency

**Fig 3:**
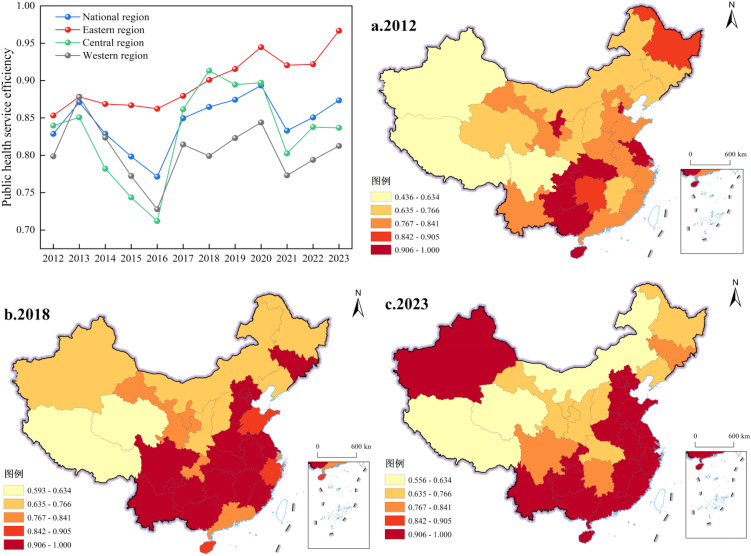
Spatial evolution pattern of public health efficiency in China, 2012–2023.

The public health service efficiency of China, as measured by the DEA-SBM model, exhibited a fluctuating upward trend during the period from 2012 to 2023, with significant differences in evolutionary trajectories across regions (see Fig 3). At the national level, the mean value of public health service efficiency increased from 0.834 in 2012 to 0.873 in 2023, representing an overall modest improvement of approximately 4.7 percentage points, during which a notable decline occurred in 2016 (falling to 0.769) followed by a subsequent recovery. Disaggregated by region, the eastern region consistently maintained the highest efficiency level, rising steadily from 0.852 in 2012 to 0.969 in 2023 with relatively limited fluctuation; the central region exhibited a “V”-shaped evolutionary pattern, with an efficiency value of 0.833 in 2012, a sharp decline to the lowest point of 0.712 in 2016, and a continuous recovery to 0.835 by 2023; the western region recorded the lowest overall efficiency level, fluctuating narrowly within the range of 0.798 to 0.844, reaching 0.810 in 2023. Notably, efficiency values across all regions demonstrated an upward trend after 2018, with the eastern region surpassing 0.90 after 2019 and continuing to climb, indicating sustained improvement in the quality of public health service provision.

The spatial distribution of public health service efficiency exhibited an evolutionary pattern transitioning from multi-core dispersion toward bipolar agglomeration in the north and south. The spatial pattern in 2012 showed that high-efficiency areas (0.906–1.000) were primarily concentrated in the northeastern region (Heilongjiang and Jilin) and the South China coastal area (Guangdong and Hainan), presenting a multi-point distribution; most provinces in the central and western regions were situated in the medium-to-low efficiency range of 0.436 to 0.766, with relatively moderate spatial disparities. In 2018, the spatial pattern underwent significant restructuring, with high-efficiency areas expanding substantially and agglomerating toward the central-southern region, forming a contiguous high-value zone centered on Hunan, Hubei, Chongqing, and Guizhou, while efficiency values in eastern coastal provinces (Jiangsu, Zhejiang, Shanghai, and Fujian) improved markedly; the northwestern region (Xinjiang, Qinghai, and Gansu) and certain southwestern provinces remained in the lowest tier of 0.593 to 0.634. By 2023, high-efficiency areas expanded further, with northwestern Xinjiang and the South China–Southwest region forming two major high-value agglomeration centers (efficiency values reaching 0.906–1.000), while eastern coastal provinces overall improved to the range of 0.767 to 0.905; low-efficiency areas contracted significantly, becoming largely confined to a small number of provinces in the northwestern interior. Overall, the spatial distribution evolved from the initial pattern of “eastern dominance and western weakness with point-like distribution” to a new pattern of “dual-core north-south agglomeration with contiguous clustering.”

### 4.3. Coupling coordination degree between carbon emission governance level and public health service efficiency

**Fig 4:**
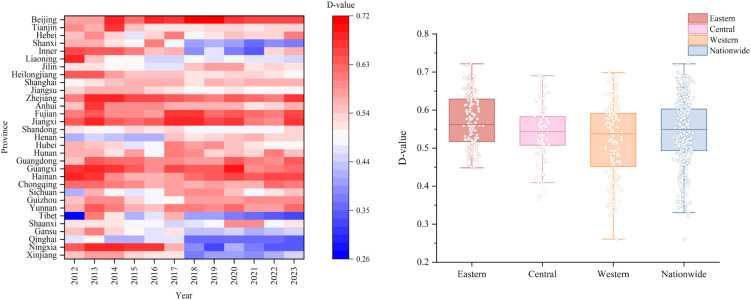
Spatiotemporal distribution characteristics of coupling coordination degree between carbon emission governance and public health efficiency in China, 2012–2023.

The coupling coordination degree between carbon emission governance level and public health service efficiency exhibited pronounced temporal volatility and interprovincial heterogeneity during the period from 2012 to 2023 (see Fig 4). As reflected in the temporal evolution heatmap, the overall national coupling coordination degree demonstrated a pattern of initial fluctuation followed by sustained improvement, with the coordination degree of most provinces maintained within the range of 0.44 to 0.63 during 2012–2017, and generally rising to the range of 0.54 to 0.72 after 2018. The consistently directional upward trend over the 12-year period indicates that the synergistic relationship between the two systems has been continuously strengthening. In terms of interprovincial relative positions, the evolution of coordination degree gaps and ranking patterns among provinces more substantively reflects the actual divergence in regional coordinated development than longitudinal comparisons against fixed thresholds — eastern coastal provinces have persistently ranked at the forefront nationally with a widening margin, while northwestern inland provinces have long remained at relatively low positions with insufficient momentum for improvement, and this entrenchment of relative patterns warrants greater attention than the absolute numerical levels. Specifically, eastern coastal provinces such as Zhejiang, Fujian, Jiangsu, and Guangdong consistently maintained high coordination levels throughout the observation period with a steadily rising trend, as reflected by a gradual deepening of color from light red to deep red; central provinces such as Hunan, Hubei, and Anhui exhibited fluctuating patterns, with a pronounced low-value period around 2016 followed by a rapid recovery; northwestern provinces such as Qinghai, Ningxia, and Xinjiang remained in a state of persistently low coordination, with the heatmap displaying conspicuous cool tones and relatively stable temporal evolution. Certain provinces such as Liaoning and Hainan registered deep red high values in 2012–2013, but subsequently experienced fluctuating declines, reflecting the instability of coordinated development.

The regional distribution of the coupling coordination degree exhibited a spatial pattern of “higher in the east and lower in the west with increasing dispersion,” yet with significant intra-regional heterogeneity. Based on the statistical characteristics of the box plots, the median coordination degree of the eastern region was approximately 0.55, with a relatively narrow interquartile range (IQR) of approximately 0.52 to 0.63 and few outliers, indicating relatively balanced intra-regional development and the highest overall level; the central region recorded a median of approximately 0.54, but with a larger box span (approximately 0.51 to 0.58) and conspicuous low-value outliers (minimum value approximately 0.40), reflecting internal differentiation; the western region had a median of approximately 0.55 but exhibited the greatest degree of dispersion, with an interquartile range spanning 0.45 to 0.59 and numerous extreme low values (minimum value approximately 0.25), indicative of highly uneven intra-regional development. At the national level, the median was approximately 0.55, but the data points were widely distributed (0.30–0.72) with a large standard deviation, indicating significant interprovincial disparities in the level of coordinated development. A comparison of the box plot distributions across the three major regions reveals that, although the differences in medians across regions are not large (all within the range of 0.54 to 0.55), the eastern region demonstrates markedly superior data concentration and stability relative to the central and western regions, with the long-tail effect and outlier phenomenon being particularly prominent in the western region.

### 4.4. Dagum decomposition of the Gini coefficient: Intra-regional, inter-regional, and transvariation components

The measurement results of regional disparities in the coupling coordination degree based on the Dagum Gini coefficient and its decomposition method indicate (see Table 5) that the overall disparity in the coupling coordination degree between carbon emission governance and public health service efficiency in China expanded significantly after 2018, with inter-regional disparities consistently constituting the primary source of overall disparity. In terms of the overall Gini coefficient, national disparities exhibited a fluctuating downward trend during 2012–2017, declining from 0.082 to 0.064, suggesting a gradual convergence in the level of coordinated development across regions; however, the overall Gini coefficient rose sharply to 0.100 in 2018 and subsequently remained at the elevated level of 0.092 to 0.099, reflecting an intensification of regional differentiation. In terms of the decomposition of disparity sources, the contribution rate of inter-regional disparities consistently ranked first, remaining stable within the range of 39.74% to 42.26% during 2012–2023, with an average contribution rate of 40.88%, indicating that the development imbalance among the three major eastern, central, and western regions is the dominant factor underlying overall disparities; the contribution rate of intra-regional disparities ranked second, accounting for 29.57% to 33.77% with an average of 31.97%; and the contribution rate of hypervariable density remained relatively stable, maintained within the range of 26.49% to 28.17%. Further examination of intra-regional disparities reveals that the intra-regional Gini coefficient of the western region consistently remained at the highest level (0.054–0.123), rising significantly to above 0.114 after 2018; intra-regional disparities in the central region were relatively smaller (0.058–0.075); and intra-regional disparities in the eastern region were the lowest (0.052–0.078). Regarding inter-regional disparities, the Gini coefficient between the eastern and western regions expanded continuously from 0.090 in 2012 to 0.114 in 2023, becoming the predominant manifestation of inter-regional disparities, while the east–central and central–west inter-regional disparities remained relatively stable. The relative stability of the contribution rate structure indicates that the composition of disparity sources exhibits persistent characteristics, and the issue of inter-regional development imbalance requires sustained long-term attention.

**Table 5:** Dagum Gini coefficient and decomposition results for the coupling coordination degree between carbon emission governance and public health service efficiency in China.

Year	Intra-regional				Inter-regional			Contribution Rate (%)		
	National	Eastern	Central	Western	East–Central	East–West	Central–West	Intra-regional	Inter-regional	Hypervariable Density
2012	0.082	0.058	0.061	0.110	0.063	0.090	0.089	33.767	39.740	26.493
2013	0.061	0.056	0.073	0.054	0.067	0.056	0.068	32.627	40.424	26.949
2014	0.072	0.068	0.061	0.073	0.076	0.072	0.074	32.882	40.271	26.847
2015	0.071	0.052	0.068	0.083	0.067	0.074	0.077	32.900	40.260	26.840
2016	0.068	0.062	0.075	0.058	0.075	0.070	0.070	31.496	41.102	27.402
2017	0.064	0.065	0.064	0.055	0.068	0.065	0.063	32.725	40.365	26.910
2018	0.100	0.078	0.072	0.114	0.080	0.118	0.111	30.980	41.412	27.608
2019	0.094	0.073	0.061	0.116	0.070	0.113	0.104	31.730	40.962	27.308
2020	0.097	0.066	0.062	0.123	0.072	0.118	0.108	31.033	41.380	27.587
2021	0.098	0.065	0.058	0.118	0.075	0.123	0.109	29.566	42.260	28.174
2022	0.092	0.064	0.061	0.117	0.070	0.109	0.100	31.696	40.982	27.322
2023	0.099	0.068	0.074	0.121	0.088	0.114	0.106	31.192	41.285	27.523

**Fig 5:**
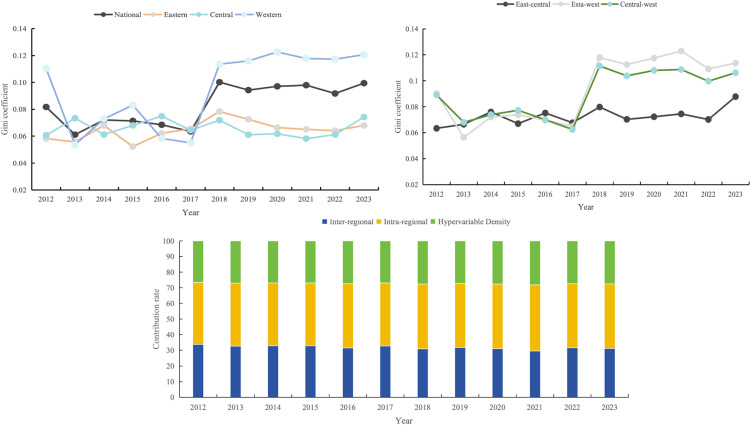
Dagum Gini coefficient and its decomposition results of the coupling coordination degree between carbon emission governance level and public health service efficiency in China.

The temporal evolution of the Gini coefficient for the coupling coordination degree exhibited pronounced stage-specific characteristics and patterns of regional heterogeneity (see Fig 5). In terms of intra-regional disparities, the Gini coefficients of all three major regions followed a convergent trend during 2012–2017, with limited fluctuation (approximately 0.05–0.08), and the national level overall developed toward equalization; 2018 marked a structural turning point, at which the Gini coefficient of the western region surged abruptly and remained persistently in the elevated range of 0.114–0.123, forming a conspicuous divergence from the eastern and central curves, while the eastern and central curves tended toward stability and cross-overlapped within the range of 0.06–0.08, presenting a pattern of “one high, two flat.” In terms of inter-regional disparities, the Gini coefficient between the eastern and western regions separated significantly upward after 2018, persistently exceeding the east–central and central–west curves by approximately 0.01–0.02 units and oscillating at elevated levels within the range of 0.109–0.123; the east–central and central–west curves remained highly synchronized throughout, fluctuating jointly around 0.07 without any significant widening of the gap. After 2018, the national Gini coefficient curve almost entirely followed the trajectories of the intra-western and east–west inter-regional curves, indicating that intra-western differentiation and the east–west inter-regional gap are the key factors driving the expansion of overall disparities. In terms of the contribution rate structure of disparity sources, the contribution rate of inter-regional disparities consistently ranked first throughout the observation period, remaining stably within the range of 39.74% to 42.26% with minimal inter-annual fluctuation; the contribution rate of intra-regional disparities ranked second, accounting for 29.57% to 33.77%; and the contribution rate of hypervariable density was relatively the lowest and most stable, maintained within the range of 26.49% to 28.17%. The contribution rate structure of the three sources remained highly stable over the 12-year period, indicating that the formation mechanism of regional disparities in the coupling coordination degree exhibits significant path dependence, and the dominant position of inter-regional development imbalance did not undergo substantive change under policy shocks.

### 4.5. Spatial evolution of coupling coordination

The results of the Global Moran’s Index test indicate that the coupling coordination degree between carbon emission governance level and public health service efficiency in China exhibits significant and continuously strengthening positive spatial autocorrelation characteristics (see Table 6). In terms of statistical testing, with the exception of 2013 and 2017, the p-values of the Global Moran’s Index for all other years were below 0.05, indicating that the spatial agglomeration pattern of the coupling coordination degree is statistically highly significant. In 2013, the Moran’s Index dropped sharply to 0.155, with the statistical significance of spatial autocorrelation temporarily disappearing, presenting a conspicuous discontinuity relative to adjacent years; a similar stage-specific low value also occurred in 2017. In terms of temporal evolutionary trends, the Global Moran’s Index exhibited an overall fluctuating upward trajectory, rising from 0.227 in 2012 to 0.497 in 2022, representing an increase of 118.9%, indicating a continuously deepening degree of spatial agglomeration; in particular, after 2018, the Moran’s Index remained stably within the elevated range of 0.393 to 0.497, with z-values consistently exceeding 3.744, demonstrating strong spatial dependence. The low value observed in 2017, accompanied by a weakening of spatial autocorrelation significance, reflects the possibility that the pattern of coordinated development among regions underwent a brief process of restructuring during that period. The overall positive and continuously strengthening spatial autocorrelation characteristics indicate that the coupling coordination degree exhibits a spatial distribution pattern of “high-value agglomeration and low-value agglomeration,” whereby provinces with high coordination levels tend to be adjacent to other provinces with high coordination levels, and provinces with low coordination levels likewise exhibit spatial agglomeration tendencies.

**Table 6:** Global Moran’s Index for the coupling coordination degree between carbon emission governance and public health service efficiency in China, 2012–2023.

Year	2012	2013	2014	2015	2016	2017	2018	2019	2020	2021	2022	2023
Moran’s I	0.227	0.155	0.311	0.421	0.320	0.128	0.410	0.440	0.393	0.447	0.497	0.343
p-value	0.012	0.059	0.002	0.000	0.002	0.067	0.000	0.000	0.000	0.000	0.000	0.001
z-value	2.273	1.562	2.883	3.786	2.961	1.501	3.744	4.003	3.811	4.033	4.475	3.197

The local spatial autocorrelation analysis reveals that the coupling coordination degree exhibits significant spatial agglomeration heterogeneity and type-specific evolutionary characteristics (see Table 7), with high-high agglomeration zones continuously expanding while low-high and high-low transition zones progressively contracting. With respect to the high-high agglomeration zones, the core areas gradually diffused from the southeastern coastal region (Guangdong, Zhejiang, Fujian, and Hainan) in 2012 toward the interior, expanding to the Yangtze River Delta and Bohai Rim regions (Shanghai, Jiangsu, Beijing, and Tianjin) in 2016, and further extending into the central and western regions (Guizhou, Hubei, and Anhui) in 2020, ultimately forming by 2023 a large-scale contiguous high-value agglomeration belt encompassing 14 provinces and covering the eastern coastal areas, the Yangtze River Economic Belt, and parts of the southwestern region, demonstrating the spatial diffusion effect of coordinated development. The low-low agglomeration zones remained persistently stable in the northwestern inland provinces (with Qinghai, Tibet, and Xinjiang consistently included), encompassing 9 provinces including Shanxi, Sichuan, and Shandong in 2012 and expanding to 11 provinces by 2023, with the characteristic of a contiguous low-value zone spanning the northwest and north China becoming increasingly pronounced. However, the number of provinces in the low-high transition zones decreased sharply from 8 in 2012 to 1 in 2023, and the number in the high-low transition zones declined from 7 in 2012 to 2 in 2023. The substantial contraction of transition zones indicates an intensification of the bipolarization trend in the spatial pattern, with the Matthew effect of “high values neighboring high values and low values neighboring low values” significantly strengthening.

**Table 7:** Changes in spatial association types for the coupling coordination degree between carbon emission governance and public health efficiency in China, 2012–2023.

Year	High–High (H–H)	Low–Low (L–L)	Low–High (L–H)	High–Low (H–L)
2012	Guangdong, Zhejiang, Fujian, Hainan, Jiangxi, Guangxi	Qinghai, Tibet, Xinjiang, Shanxi, Sichuan, Shandong, Gansu	Jilin, Beijing, Jiangsu, Shanghai, Guizhou	Yunnan, Hebei, Heilongjiang, Ningxia, Chongqing
2016	Fujian, Hainan, Tianjin, Jiangsu, Shanghai, Zhejiang, Guangdong, Jiangxi, Beijing, Anhui, Guangxi, Shandong	Qinghai, Xinjiang, Jilin, Gansu, Sichuan, Liaoning, Shanxi, Tibet, Inner Mongolia, Shaanxi	Hunan, Guizhou, Ningxia, Chongqing	Ningxia, Chongqing, Heilongjiang, Hebei
2020	Hainan, Fujian, Guangdong, Guangxi, Beijing, Jiangxi, Zhejiang, Guizhou, Anhui, Hubei, Tianjin	Tibet, Qinghai, Xinjiang, Ningxia, Gansu, Liaoning, Inner Mongolia	Shandong	Jilin, Hainan, Shaanxi, Hebei
2023	Guangdong, Guangxi, Hainan, Fujian, Chongqing, Hunan, Tianjin, Jiangxi, Shanghai, Zhejiang, Shandong, Beijing, Guizhou	Qinghai, Tibet, Inner Mongolia, Ningxia, Shaanxi, Liaoning, Gansu, Xinjiang	Hubei	Sichuan, Henan

### 4.6. Influencing factors

#### 4.6.1. Level of economic development.

The effect of per capita GDP on the coupling coordination degree exhibits significant regional heterogeneity (see Table 8). At the national level, the coefficient is 0.019, which fails to pass the significance test (p > 0.1). Disaggregated by region, the coefficient for the eastern region is 0.076 (p < 0.01), indicating a significant positive marginal effect of economic development level on the coupling coordination degree in the east; the coefficient for the central region is −0.079 (p < 0.01), representing a significant negative effect; and the coefficient for the western region is −0.012, which does not reach statistical significance. It is thus evident that the effect of economic development level on the coupling coordination degree is inconsistent across regions, with significant inter-regional differences.

**Table 8:** Results of the two-way fixed-effects Tobit model for influencing factors.

	(1)	(2)	(3)	(4)
VARIABLES	National	Eastern	Central	Western
GDPpc	0.019	0.076***	−0.079***	−0.012
	−0.019	−0.018	−0.019	−0.043
UrbR	−0.306*	0.000	0.063	−0.311
	−0.180	−0.001	−0.578	−0.419
Pop65+	0.019	−0.017	−0.024	0.048*
	−0.014	−0.013	−0.021	−0.028
R&DInt	0.045**	−0.024	0.185***	0.110*
	−0.022	−0.017	−0.037	−0.057
PopDen	−0.080***	−0.036	−0.026	−0.139***
	−0.030	−0.048	−0.048	−0.051
sigma_u	0.101***	0.073***	0.072***	0.096***
	−0.014	−0.018	−0.020	−0.024
sigma_e	0.090***	0.053***	0.056***	0.116***
	−0.003	−0.003	−0.004	−0.007
Constant	1.154***	1.011***	0.475	1.252***
	−0.133	−0.157	−0.347	−0.259
Observations	372	132	96	144
Number of id	31	11	8	12

Standard errors in parentheses.

*** p < 0.01, ** p < 0.05, * p < 0.1.

#### 4.6.2. Social structure.

The coefficient of urbanization rate at the national level is −0.306, which is significantly negative at the 10% significance level; however, the standard error reaches 0.180, indicating limited estimation precision. The region-specific coefficients are −0.000 for the eastern region, 0.063 for the central region, and −0.311 for the western region, none of which pass the significance test. This indicates that the estimation of this variable in the overall sample is subject to considerable uncertainty. Therefore, within the present analytical framework, no statistically significant evidence is found for a direct effect of urbanization rate on the coupling coordination degree, and its underlying mechanism warrants further investigation.

#### 4.6.3. Demographic structure.

The coefficient of the aging population proportion (std_x3) at the national level is 0.019, which does not reach statistical significance. Disaggregated by region, the coefficients for the eastern region (−0.017) and the central region (−0.024) are both statistically insignificant, while the coefficient for the western region is 0.0481, which is significantly positive at the 10% significance level. Only in the western region does this variable exhibit a statistically significant positive association with the coupling coordination degree, with no significant effects observed in the remaining regions. This indicates that the effect of aging on the coupling coordination degree is not linearly consistent, but rather exhibits directional divergence due to differences in regional resource conditions and system carrying capacity. The significant positive effect in the western region stands in sharp contrast to the non-significant negative tendency observed in the eastern and central regions, and the specific underlying mechanism warrants further analysis.

#### 4.6.4. Environmental regulation.

The coefficient of R&D expenditure intensity (x4) at the national level is 0.045 (p < 0.05), indicating that technological innovation investment exerts a significant positive promotional effect on the coupling coordination degree. Disaggregated by region, the coefficient is largest for the central region (0.185, p < 0.01), followed by the western region (0.110, p < 0.1), while the coefficient for the eastern region is −0.024, which does not reach statistical significance. The marginal effect of R&D investment on the coupling coordination degree exhibits a gradient characteristic of increasing magnitude from east to west, reflecting the diminishing marginal returns to technological innovation under the already high baseline governance level of the eastern region, as well as the reality that technological investment in the central and western regions remains at a critical stage of gap-filling.

#### 4.6.5. Population density.

The coefficient of the logarithm of urban population density at the national level is −0.080 (p < 0.01), representing a significant negative effect. Disaggregated by region, the coefficients for the eastern region (−0.036) and the central region (−0.026) both fail to reach statistical significance; the coefficient for the western region is −0.139 (p < 0.01), exhibiting the most pronounced negative effect. This indicates that spatial population agglomeration in the western region has failed to generate agglomeration economic benefits; instead, the expansion of the public service supply radius and the rising per capita infrastructure costs have formed a significant inhibitory effect on the overall level of coordination, while this effect has yet to reach statistical significance in the eastern and central regions.

### 4.7. Robustness checks

To examine the robustness of the baseline regression results with respect to spatial dependence among provinces, this study re-estimates the national sample using a two-way fixed effects model with Driscoll-Kraay standard errors. Driscoll-Kraay standard errors are capable of simultaneously correcting for the disturbances arising from cross-sectional dependence (i.e., spatial dependence among provinces), serial correlation, and heteroskedasticity in panel data parameter estimation, and represent a well-established econometric approach for addressing spatial correlation in panel data (Driscoll & Kraay, 1998). As shown in Table 9, following the introduction of Driscoll-Kraay standard errors, the coefficient directions of all core explanatory variables remain highly consistent with the baseline Tobit regression results. In addition, given that the two-way fixed effects Tobit model is subject to the incidental parameters problem in handling individual effects, this study further re-estimates the national sample using a Random Effects Tobit Model to examine the robustness of the baseline regression conclusions with respect to model specification, with the directions of all variables consistent with those of the baseline regression. To further mitigate potential reverse causality bias between the explanatory variables and the coupling coordination degree, this study incorporates all explanatory variables lagged by one period into the Random Effects Tobit Model for re-estimation. The results indicate that the coefficient directions of all core significant variables remain highly consistent with those of the baseline regression, with no substantive shift in the magnitude of the coefficients; moreover, the coefficients of variables that were insignificant in the baseline regression do not yield significant results contrary to the baseline direction following the lag treatment. The foregoing results demonstrate that the core conclusions of the baseline regression possess satisfactory robustness.

**Table 9:** Robustness check results.

	(1)	(2)	(3)	(4)
VARIABLES	Baseline Tobit	DK Fixed Effects	Random Tobit	One-period lag
GDPpc	0.019	0.004	0.013	0.006
	−0.019	0.015	0.016	0.017
UrbR	−0.306*	−0.769**	−0.569**	−0.414**
	−0.180	0.301	0.167	0.174
Pop65+	0.019	0.002	−0.004	−0.006
	−0.014	0.008	0.012	0.012
R&DInt	0.0454**	0.072***	0.080***	0.058***
	−0.022	0.020	0.019	0.021
PopDen	−0.0798***	−0.123**	−0.094**	−0.062**
	−0.030	0.044	0.030	0.031
Constant	1.154***	1.453***	1.223***	1.056***
	−0.133	0.249	0.140	0.144
Observations	372	372	372	341
Number of id	31	31	31	31

Standard errors in parentheses.

*** p < 0.01, ** p < 0.05, * p < 0.1.

## 5. Discussion and recommendations

### 5.1. Discussion

#### 5.1.1. Sustained and gradual improvement in coordination level, with policy transition pains and institutional time lags constraining the deepening of synergistic progress.

During the period from 2012 to 2023, the coupling coordination degree between carbon emission governance and public health efficiency in China exhibited an overall evolutionary pattern of initial fluctuation followed by a sustained upward trend, with the coordination degree of most provinces rising from the range of 0.44–0.63 to the range of 0.54–0.72. The consistently directional improvement over 12 years indicates that the coordinated advancement of the “dual carbon” targets and the Healthy China strategy has preliminarily driven a virtuous interaction between the two systems. Nevertheless, the coordination degree of most provinces has yet to break through the threshold of intermediate coordination, the depth of inter-system synergy remains limited, and the pattern of interprovincial differentiation has not undergone fundamental restructuring. Examining the temporal trajectory, the coordination degree trough in 2016 — during which the mean public health efficiency value dropped sharply from 0.834 to 0.769, with the central region declining to 0.712 — concentrated the manifestation of three structural tensions. Drawing on existing theoretical frameworks, the literature, and plausible contributing factors [57,58], the study identifies the following three potential mechanisms: first, the input-output time lag arising from the intensive implementation of healthcare reform policies such as hierarchical diagnosis and treatment systems and family physician contracting; second, the competitive allocation between environmental protection and healthcare expenditure triggered by the transmission of the rigid carbon emission constraints of the 13th Five-Year Plan to local fiscal systems; and simultaneously, the paradoxical pattern of “increasing bed numbers accompanied by declining efficiency” formed by the concurrent outflow of medical personnel and persistently low facility utilization rates in the central region. The overall recovery in coordination degree after 2018 is closely associated with the policy synergy generated by the deepening of ecological civilization institutional reform and the comprehensive launch of the Healthy China strategy, indicating that the degree of integration of cross-sectoral policy frameworks constitutes a key institutional variable driving the sustained improvement of provincial coordination levels.

#### 5.1.2. Inter-regional disparities dominate overall divergence, with factor endowment differences and fiscal system logic jointly entrenching the east-west divide.

Inter-regional disparities have consistently constituted the dominant source of overall disparities in the coupling coordination degree, with structural imbalances characterized by “deepening east-west divide and increasing intra-western dispersion” intensifying. The decomposition results of the Dagum Gini coefficient indicate that the contribution rate of inter-regional disparities remained stably within the range of 39.74%–42.26% throughout the observation period, and after 2018, the Gini coefficient between the eastern and western regions expanded continuously from 0.090 to 0.114, while the intra-western Gini coefficient surged from 0.110 to the elevated range of 0.114–0.123. This pattern is consistent with the inherent differences in factor endowments between the eastern and western regions: the comprehensive level of carbon emission governance in the eastern region persistently remained at a high level throughout the observation period and continued to improve, while the western region has long lingered in the low-value range, with the mean gap between the two systems providing direct empirical support for the continuous expansion of the inter-regional Gini coefficient. From the perspective of the fiscal system, the fiscal self-sufficiency rate of western provinces is relatively low, and the fragmented allocation of central transfer payments may be insufficient to effectively drive systematic improvements in public health service efficiency; however, given the limited availability of direct fiscal data, the precise estimation of the magnitude of this mechanism awaits further deepening in subsequent research. The intensification of carbon emission governance policies since 2018 has also produced markedly asymmetric shocks to the industrial structures of different regions — the eastern region completed industrial green transformation first, advancing carbon emission reduction and economic benefits in tandem; the central and western regions remain highly dependent on heavy industry and resource-based industries, experiencing more severe structural adjustment pains under emission reduction constraints, which indirectly weakens the resource allocation capacity of local governments in the healthcare sector and further entrenches the coordination degree gap between the eastern and western regions.

#### 5.1.3. Sustained strengthening of positive spatial autocorrelation, with factor mobility mechanisms and institutional connectivity effects jointly driving agglomeration polarization.

The statistical characteristics of continuously strengthening spatial agglomeration are pronounced, with the Global Moran’s Index rising from 0.227 in 2012 to 0.497 in 2022, representing an increase of 118.9%. The local spatial autocorrelation results indicate that the high-high agglomeration zones expanded from 4 provinces in 2012 to 14 in 2023, the low-low agglomeration zones likewise expanded from 9 to 11 provinces, and transition-type provinces contracted substantially, with the bipolarization pattern clearly manifested at the data level. The continuous expansion of high-value agglomeration zones demonstrates a degree of temporal alignment with the advancement of cross-administrative-region collaborative policies in areas such as the Yangtze River Delta and Pearl River Delta, while the low-value locked zones have long been concentrated in the northwestern inland provinces, corresponding to the persistently low performance of these provinces in terms of carbon emission governance level and public health efficiency. The two stage-specific low values of the Moran’s Index in 2013 and 2017 can be interpreted in conjunction with major contemporaneous policy milestones: in 2013, the Third Plenary Session of the 18th Central Committee of the Communist Party of China deliberated and adopted the Decision on Several Major Issues Concerning Comprehensively Deepening Reform, incorporating ecological civilization construction into the “Five-in-One” overall layout for the first time; during the policy transition period in which the implementation details awaited clarification at the provincial level, the interprovincial coordination gap experienced a brief narrowing. In 2017, the comprehensive implementation of the 13th Five-Year Plan for Poverty Alleviation directed large-scale targeted fiscal resources toward underdeveloped county-level areas, which in the short term raised the coordination levels of low-value provinces, compressed the spatial gap among provinces, and produced a stage-specific homogenization effect. The foregoing patterns indicate that well-documented targeted policy interventions possess realistic potential to temporarily disrupt spatial polarization in the short term; however, the homogenization effect is difficult to sustain, and once the policy dividend period concludes, the market logic of factor mobility and the structural constraints of endowment differences will drive spatial agglomeration back onto a strengthening trajectory, underscoring the imperative of designing institutional frameworks for long-term regional coordination policies.

#### 5.1.4. Significant regional heterogeneity in associated factors, with divergent economic development pathways and differential marginal returns to technological innovation jointly shaping the coordination pattern.

The region-specific results of the two-way fixed effects Tobit regression reveal that the direction and magnitude of the associations between various factors and the coupling coordination degree differ significantly across the eastern, central, and western regions, reflecting the fact that the associative logic between these factors and coordinated development is not uniform across different stages of development and resource endowment conditions. The level of economic development (per capita GDP) exhibits a significant positive association with the coupling coordination degree in the eastern region (β = 0.076, p < 0.01), yet a significant negative association in the central region (β = −0.079, p < 0.01), while the coefficient for the western region is −0.012 and statistically insignificant. Given that the aforementioned associations represent correlations rather than causal relationships and may partially reflect the confounding influence of omitted variables, an alternative explanation nonetheless warrants attention: provinces in the central region with higher per capita GDP tend also to be those with a more concentrated heavy industrial base and higher costs of industrial structural transformation, and the structural contradiction between industrial carbon emission intensity and healthcare resource allocation may jointly suppress the coupling coordination degree of these provinces, thereby producing a statistically negative association between economic development level and coordination degree. Furthermore, provincial-level omitted variables such as biases in fiscal expenditure structure and differences in the stringency of environmental regulation enforcement may also confound this association. R&D expenditure intensity is significantly positively associated with the coupling coordination degree at the national level (β = 0.045, p < 0.05); disaggregated by region, the association is most pronounced in the central region (β = 0.185, p < 0.01), followed by the western region (β = 0.110, p < 0.1), with no significant association observed in the eastern region, exhibiting a gradient characteristic of increasing magnitude from east to west. This indicates that a strong positive association persists between technological innovation investment and improvements in coordination degree in the central and western regions, making it one of the factors most closely associated with the coordination level of the two systems in the central and western regions at the present stage. Urban population density exhibits a significant negative association with the coupling coordination degree both nationally (β = −0.080, p < 0.01) and in the western region (β = −0.139, p < 0.01), with no significant associations observed in the eastern and central regions; this association is consistent with the relatively weak public service supply capacity in the western region. The urbanization rate and the aging population proportion fail to reach statistical significance in most regions, and their net associations with the coupling coordination degree remain indeterminate within the present research framework. The foregoing results collectively indicate that economic development level and technological innovation investment are the factors most prominently associated with coordinated development across regions, and that the magnitude of these associations varies significantly with regional development conditions, such that differentiated factor allocation strategies carry important reference value for enhancing regional coordination levels.

### 5.2. Recommendations

#### 5.2.1. Establishing cross-sectoral policy sequencing and coordination mechanisms to alleviate resource misallocation induced by institutional time lags.

Strengthening cross-sectoral policy sequencing coordination and fiscal synergy management should be prioritized to break through the resource misallocation caused by institutional time lags and policy conflicts. The study finds that the coupling coordination degree exhibited a pronounced trough during periods of intensive concurrent implementation of carbon emission constraint policies and healthcare reform policies, most notably in the central region, and that the coordination degree promptly recovered following an increase in the degree of policy framework integration, indicating that temporal conflicts among cross-sectoral policies constitute an important associated factor constraining the coordinated advancement of the two systems. It is recommended that a linkage-based budget protection mechanism for environmental and healthcare expenditure be established at the provincial level, with a clearly defined rigid floor for public health expenditure, so as to prevent the transmission of emission reduction constraints to local fiscal systems from generating a crowding-out effect on healthcare investment. Incorporating reductions in carbon emission intensity and improvements in public health service efficiency into a unified official performance evaluation system would employ institutional constraints to incentivize local governments to proactively resolve the resource competition between the two systems. With respect to the output time lag of healthcare reform policies, it is recommended that efficiency evaluation mechanisms be embedded in newly added primary-level healthcare investment projects, with regular tracking of input-output changes and timely initiation of resource adjustment procedures for projects exhibiting pronounced time lags, so as to prevent sustained investment from depressing overall efficiency levels. From the perspective of institutional design, the coordinated planning of environmental governance and public health policies should be incorporated into top-level arrangements, replacing ex post remedial resource redistribution with ex ante institutional coordination, so as to fundamentally compress the interference space of institutional time lags on the coordination process.

#### 5.2.2. Constructing a differentiated regional coordination policy framework to unblock institutional bottlenecks in the east-west divide.

Region-specific and category-specific policy implementation grounded in regional heterogeneity characteristics should be pursued, leveraging differentiated interventions to narrow the divergence in coordinated development across the three major regions. Inter-regional disparities have consistently been the dominant source of overall disparities, with the east-west divide continuing to widen after 2018 and intra-western differentiation simultaneously intensifying. The heterogeneous effects of influencing factors likewise indicate that the core constraints on coordinated development differ fundamentally across regions, making it imperative to construct differentiated precision intervention frameworks based on the core constraints of each region, replacing large-scale resource accumulation with structural policy reorientation. The policy focus of the eastern region should shift from incremental investment toward the optimization of existing stock efficiency, exploring market-oriented collaborative pathways between carbon governance and public health services to establish institutional models replicable in the central and western regions. The central region should prioritize the promotion of green structural adjustment in its industrial composition, establishing a linkage mechanism between emission reduction targets and improvements in healthcare efficiency, preventing singular emission reduction constraints from compressing local healthcare fiscal space, and gradually resolving the structural contradiction between economic expansion and improvements in coordination degree. Given the pronounced intra-regional differentiation problem in the western region, it is recommended that transfer payment funds be concentrated toward county-level units with weak coordination capacity, while simultaneously promoting institutional transplantation and technology transfer from the eastern to the western region, replacing mere financial transfers with capacity building so as to fundamentally enhance the endogenous coordination capacity of western provinces.

#### 5.2.3. Activating regional spatial spillover effects and interrupting the self-reinforcing cycle of low-value agglomeration.

A regional collaborative policy system oriented toward the dual objectives of activating high-value spillovers and blocking low-value lock-in should be constructed, replacing isolated provincial-level policy efforts with collective action mechanisms. Spatial agglomeration continued to intensify throughout the observation period, with high-value and low-value agglomeration zones expanding simultaneously, transition-type provinces contracting substantially, and the bipolarization pattern trending toward entrenchment; moreover, the two targeted policy intervention nodes demonstrated a high degree of temporal alignment with the stage-specific low values of the Moran’s Index, indicating that targeted policy investment possesses the potential to temporarily disrupt spatial polarization in the short term, but the homogenization effect is difficult to sustain once the policy dividend period concludes, and spatial agglomeration promptly returns to a strengthening trajectory. With regard to activating high-value spillovers, high-coordination provinces in the Yangtze River Delta, Pearl River Delta, and similar regions should be encouraged to deepen cross-provincial coordination mechanisms, engaging in substantive cooperation on issues such as green technology sharing, the construction of regional environmental health data platforms, and cross-provincial public health collaborative governance, so as to convert the advantages of institutional connectivity into systemic public goods capable of radiating to surrounding areas and orderly expanding the spatial coverage of high-value agglomeration zones. With regard to blocking low-value reinforcement, precision-targeted assistance should be implemented for northwestern inland provinces that have long remained in low-low agglomeration zones, encouraging high-coordination provinces to continuously export healthcare service management experience and green technology support, replacing traditional aid-construction models with trustee-based cooperative arrangements so as to sustainably enhance the local operational capacity of recipient areas. Institutional capacity building and long-term technical support mechanisms should replace stage-specific resource injections, gradually improving the endogenous coordination capacity of low-value agglomeration zones and interrupting the self-reinforcing cycle of low-value agglomeration at the institutional level.

#### 5.2.4. Implementing region-specific precision factor allocation and activating the maximum associative benefits of relevant factors through differentiated policy instruments.

Differentiated factor allocation priorities should be formulated, replacing nationally uniform deployment with precision policy instruments. The empirical results indicate that the direction of association between economic development level and coordination degree differs fundamentally between the eastern and central regions, that the positive association of R&D investment intensity increases progressively from east to west, and that urban population density exerts a significant inhibitory effect on the coordination degree of the western region; the core associated factors for coordinated development are not uniform across regions, making differentiated policy implementation a necessary prerequisite for enhancing policy effectiveness. At the level of policy implementation, given that the economic development level of the eastern region has already demonstrated a stable positive association with the coordination degree, the policy focus should shift toward the sustained optimization of economic growth quality, promoting the incorporation of healthcare institution energy consumption management into the carbon trading accounting framework, and using market-based incentives to guide the green and intensive utilization of healthcare resources, thereby forming a virtuous pattern in which economic growth and improvements in the coordination of the two systems advance in tandem. In the central region, the negative association between economic growth and coordination degree reflects the fact that economic expansion at the present stage has yet to effectively drive the simultaneous improvement in the efficiency of both systems; the priority should therefore be placed on promoting the clean transformation of the industrial structure, establishing a provincial industrial guidance fund doubly linked to emission reduction targets and improvements in healthcare efficiency, and giving priority credit and fiscal support to industrial projects meeting green standards, so as to gradually resolve the structural contradiction between economic growth and improvements in coordination degree. In the western region, where the positive association between R&D investment intensity and coordination degree is most prominent, technological innovation investment should be treated as the priority policy lever for driving improvements in coordination degree; national key science and technology projects should prioritize allocating grants to western research institutions, and eastern enterprises should be encouraged to establish green technology joint laboratories in the western region through market-based approaches. Simultaneously, in response to the significant inhibitory effect of urban population density on the coordination degree of the western region, the maintenance of public health service coverage rates should be established as a binding prerequisite for advancing urbanization, with the simultaneous promotion of grid-based layout of rural healthcare services and the construction of digital telemedicine access, so as to fundamentally alleviate the structural inhibition of population agglomeration on the coordination level of the western region.

## 6. Research limitations

Supported by a systematic analytical framework and multidimensional empirical methods, this study presents a relatively comprehensive account of the spatiotemporal evolutionary patterns and associated factors of the coupling coordination degree between carbon emission governance and public health efficiency in China; however, several limitations remain. First, at the level of causal identification, the associations identified by the two-way fixed effects Tobit regression between various factors and the coupling coordination degree represent correlational rather than strictly causal relationships; endogeneity issues including reverse causality, omitted variable bias, and measurement error have not been fully addressed, some mechanistic interpretations carry a degree of speculation, and the potential pathways — such as industrial structure and fiscal allocation — underlying the negative association between per capita GDP and coordination degree in the central region await more precise identification. Second, at the level of indicator construction, both the entropy weight method and the DEA-SBM model rely on the selection of proxy indicators, and different combinations of indicators may affect the absolute level of composite scores; the ten-tier classification thresholds for the coupling coordination degree are set with reference to existing literature and involve a degree of subjectivity, and potential measurement errors in the composite indicator system may also introduce a degree of interference into the results. Third, at the spatial scale level, adopting provincial-level administrative units as the unit of analysis facilitates the presentation of macro-regional patterns, but provincial-level data may obscure heterogeneous characteristics between urban and rural areas within provinces and among urban agglomerations, and different causal pathways are likewise difficult to effectively distinguish at the provincial level. Future research may build upon these limitations in the following directions: first, employing instrumental variable methods or quasi-natural experimental designs based on policy interventions to rigorously test the causal mechanisms of key associated factors, and exploring data-driven methods for delineating coupling coordination degree thresholds to enhance the objectivity of classification results; additionally, extended analyses at the prefecture-city or county level may be conducted to capture local disparities in the coordinated development of carbon emission governance and public health efficiency at a finer spatial granularity, thereby providing more operationally actionable empirical evidence for the precise targeting of policies.

## Supporting information

S1 Data Raw data used in this study. (XLSX)
